# Collagen IV outperforms alternative ECM coatings to preserve human neural progenitor properties on electrically conductive neural interfaces

**DOI:** 10.1038/s41598-026-57453-x

**Published:** 2026-06-10

**Authors:** Lin Li, Ethan Khanh Pham, Rylee Phoebe Chung, Jindi Sun, Shang Song

**Affiliations:** 1 Department of Biomedical Engineering, The University of Arizona, Tucson, AZ 85721 USA; 2 Departments of Neuroscience GIDP, Materials Science, Bio5 Institute, The University of Arizona, Tucson, AZ 85721 USA

**Keywords:** Human neural progenitor cells, Collagen IV, Extracellular matrix, Conductive interface, Neural engineering, Biological techniques, Biotechnology, Engineering, Materials science

## Abstract

Human neural progenitor cells (hNPCs) are promising candidates for neural repair, however, their in vitro expansion commonly relies on Matrigel, a tumor-derived and xenogeneic matrix that limits translational applicability. In this study, we evaluated defined extracellular matrix (ECM) coatings, including poly-L-ornithine (PLO), laminin, PLO/laminin, and collagen IV, as alternatives to Matrigel for hNPC culture under conventional and electrically stimulated conditions. We found that collagen IV consistently supported hNPC viability, proliferation, and maintenance of progenitor markers Nestin and SOX1 at levels comparable to Matrigel and superior to other chemically defined substrates. Enhanced ERK/MAPK signaling on collagen IV suggested integrin-mediated adhesion as a key mechanism underlying sustained cell survival. When applied to conductive indium tin oxide (ITO) neural interfaces, ECM coatings increased surface hydrophilicity without compromising electrical conductivity. Notably, collagen IV coated ITO maintained high hNPC viability and progenitor identity under uniform electrical stimulation. These findings identify collagen IV as a defined, electrically compatible ECM coating for conductive neural interfaces in translational neural engineering.

## Introduction

Human neural progenitor cells (hNPCs) possess the ability to differentiate into major cell types of the nervous systems, including neurons, astrocytes and oligodendrocytes. Therefore, hNPCs have been widely applied in the treatment of neurological diseases^1–3^. However, hNPCs exhibit poor adhesion to standard tissue culture surface, necessitating surface modification to support cell attachment and survival^4^. In addition to providing mechanical anchorage, surface coatings play a critical role in regulating cell morphology, proliferation, and lineage commitment by mediating cell–extracellular matrix interactions^5,6^.

Matrigel is one of the most widely utilized surface coating materials for stem cell culture. Derived from the basement membrane of the Engelbreth–Holm–Swarm (EHS) sarcoma, it contains key extracellular matrix (ECM) components, including laminin, collagen IV, entactin, as well as a variety of growth factors and bioactive peptides^7^. These constituents collectively provide a biomimetic microenvironment that supports cell adhesion, proliferation, and differentiation. Furthermore, the structural and biochemical cues offered by Matrigel enhance stem cell behavior by promoting cellular attachment and lineage specification^8^. Despite its widespread use, the applicability of Matrigel remains limited due to its complex, poorly defined, and batch-to-batch variable composition. The use of animal-derived ECM materials poses a risk of introducing xenogenic contaminants, thereby potentially compromising the therapeutic applicability of cells or tissues expanded under such conditions. Notably, viral contaminants, most prominently lactate dehydrogenase-elevating virus (LDHV), have been detected in multiple batches of animal-derived ECM products, including Matrigel^9^. As a result, numerous alternative substrates have been developed for stem cell culture.

As the main component in Matrigel and ECM, laminin is intimately involved in cell adhesion, migration and neurite growth^10^. Previous studies reported that laminin enhances total neurite length of neurons derived from cortical neural stem/precursor cells^11^. Collagen IV is another commonly used coating material for various cells, and it constitutes around 30% of the Matrigel. Accumulating studies indicate that Collagen IV facilitates the migration and transplantation of embryonic stem cells through α2β1 integrin-mediated actin remodeling^12^. It was reported that collagen IV promotes the differentiation of NPCs in cortical cell culture^13^. On the other hand, as a synthetic coating substrate, Poly-L-ornithine (PLO) facilitates cell adhesion through its positively charged amino acid polymer, while it primarily supports cell attachment through electrostatic interactions but lacks integrin-mediated signaling required for stable adhesion and long-term cell maintenance^14^.

In addition to biochemical and structural cues provided by extracellular matrix substrates, electrical stimulation (ES) has been widely applied as a therapeutic modality to enhance neural repair and functional recovery in clinical and translational settings^15–20^. In both spinal cord injury (SCI) and peripheral nerve injury (PNI), ES has been shown to promote axonal regeneration, accelerate reinnervation, and improve sensorimotor outcomes by modulating neuronal excitability, ion channel activity, and neurotrophic signaling pathways^20–23^. Increasing evidence further suggests that ES can directly influence the fate and maturation of neural progenitor cells, modulating neural progenitor cell behavior, including effects on differentiation and neurite extension under specific conditions^24–26^. Given the growing clinical use of electrically active implants and stimulation-assisted rehabilitation strategies, integrating defined, implant-compatible substrates with conductive interfaces capable of supporting hNPCs under ES conditions is of particular translational importance.

In this study, we systematically evaluated commonly used coating substrates, including PLO, laminin, PLO/laminin, and collagen IV, for hNPC culture in comparison with Matrigel. Substrate-dependent effects on cell viability, progenitor marker expression, and ERK/MAPK signaling were assessed using complementary biochemical and imaging-based analyses. Given the clinical relevance of electrical stimulation in neural repair and the increasing use of conductive biomaterials, these coatings were further evaluated on conductive ITO interfaces under defined electrical stimulation conditions. Across all assays, collagen IV consistently supported hNPC viability and preserved canonical progenitor markers at levels comparable to Matrigel and superior to other defined coatings. Notably, collagen IV maintained stable cell attachment and progenitor identity under electrical stimulation, with viability modestly exceeding that observed on Matrigel-coated conductive substrates. Collectively, these findings identify collagen IV as a defined, implant-compatible extracellular matrix that is well suited for both conventional and electrically active neural progenitor culture systems.

## Results

### Substrate-dependent regulation of hNPC viability and phenotype maintenance

**Fig. 1:**
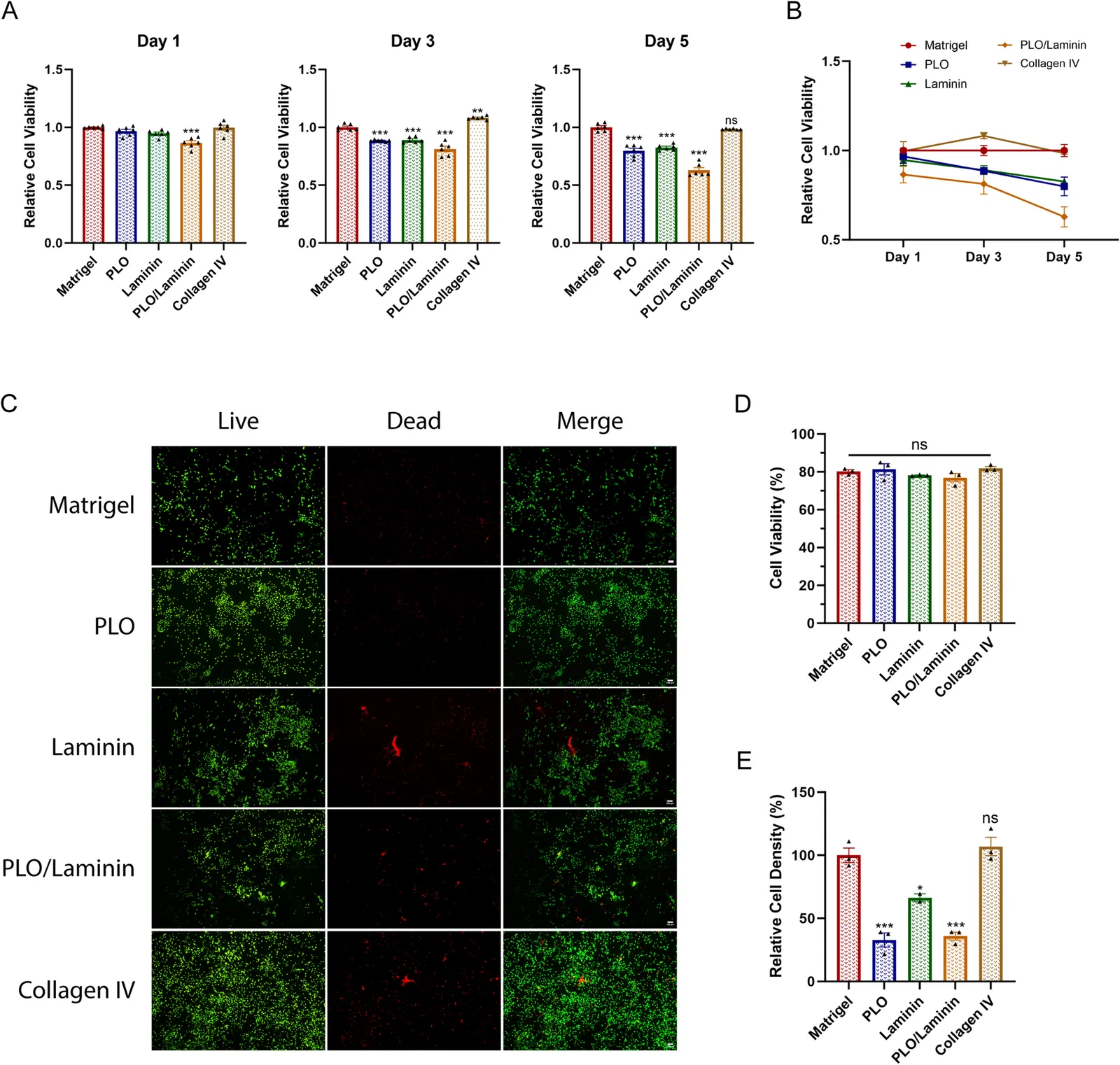
Effects of different coating substrates on hNPC viability. (**A**,** B**) Relative cell viability of hNPCs cultured on Matrigel, poly-L-ornithine (PLO), laminin, PLO/laminin, and collagen IV on day 1, day 3, and day 5, as assessed by Alamar Blue assay. (**C**) Representative live/dead staining images of hNPCs cultured on the indicated substrates. Live cells are shown in green and dead cells in red. (**D**) Quantification of relative cell viability based on live/dead staining. (**E**) Relative cell density of hNPCs cultured on different substrates. Data are presented as mean ± SEM. Statistical significance is indicated as *p < 0.05, **p < 0.01, ***p < 0.001; ns, not significant. Scale bars = 100 μm.

To evaluate the effects of different coating substrates on hNPC survival, Alamar Blue assays and live/dead staining were performed. As shown in Fig. 1A and **B**, hNPCs cultured on PLO, laminin, and PLO/laminin exhibited a gradual decline in relative viability over the 5-day culture period compared with those cultured on Matrigel. In contrast, collagen IV supported hNPC viability to a level comparable to Matrigel throughout the culture duration, indicating its favorable biocompatibility. Live/dead staining was conducted to further assess cell survival (Fig. 1C). Quantitative analysis revealed that more than 75% of cells remained viable after 5 days on all tested substrates, with no statistically significant differences observed among groups (Fig. 1D, *p* > 0.05). Notably, hNPCs cultured on collagen IV displayed a markedly higher cell density relative to the other substrates (Fig. 1E), suggesting that collagen IV may promote enhanced cell viability while maintaining high cell viability.

**Fig. 2:**
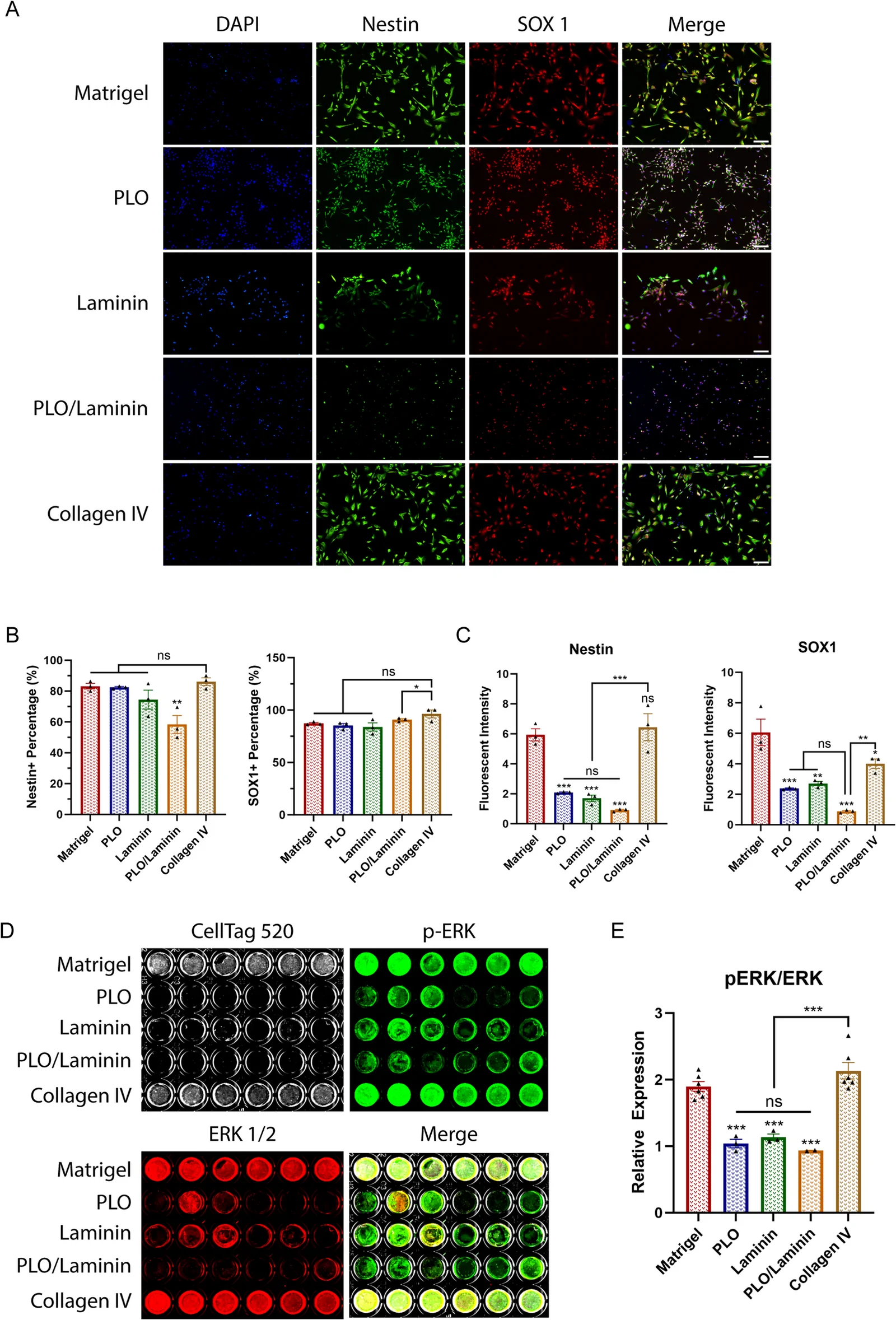
Substrate-dependent maintenance of hNPC progenitor phenotype and ERK/MAPK signaling activity. (**A**) Representative immunofluorescence images of hNPCs cultured on Matrigel, poly-L-ornithine (PLO), laminin, PLO/laminin, and collagen IV, stained for nuclei (DAPI, blue), Nestin (green), and SOX1 (red). (**B**) Quantification of the percentage of Nestin⁺ and SOX1⁺ hNPCs on different substrates. (**C**) Quantitative analysis of fluorescence intensity of Nestin and SOX1. (**D**) Representative in-cell western blot images showing CellTag 520 (total cell normalization), phosphorylated ERK (p-ERK), total ERK1/2 in hNPCs cultured on different substrates. (**E**) Quantification of p-ERK normalized to total ERK (p-ERK/ERK). Data are presented as mean ± SEM. Statistical significance is indicated as *p < 0.05, **p < 0.01, ***p < 0.001; ns, not significant. Scale bars = 100 μm.

Immunofluorescence staining was performed to examine whether hNPCs maintained their progenitor characteristics after 5 days of culture on different substrates (Fig. 2A). Nestin and SOX1, two canonical markers of hNPCs, were robustly expressed in cells cultured on Matrigel, PLO, laminin, and collagen IV. In contrast, reduced progenitor marker expression on PLO/laminin was accompanied by decreased cell adhesion and density, suggesting altered cell state. Quantitative analysis confirmed that the percentage of Nestin⁺ and SOX1⁺ cells was comparable among the Matrigel, PLO, laminin, and collagen IV groups, whereas the PLO/laminin group showed significantly lower SOX1 expression compared with collagen IV (Fig. 2B). Despite the widespread expression of progenitor markers across most groups, marked differences in fluorescence intensity and cellular morphology were observed. Cells cultured on Matrigel and collagen IV exhibited stronger Nestin and SOX1 fluorescence intensity and larger expression areas than those cultured on PLO, laminin, or PLO/laminin (Fig. 2C). Morphologically, hNPCs on Matrigel and collagen IV displayed a well-spread, multipolar phenotype characteristic of healthy progenitor cells. In contrast, cells on PLO and laminin appeared less extended, while those on PLO/laminin adopted a more rounded morphology, indicative of weaker substrate–cell interactions. To explore potential signaling mechanisms underlying these substrate-dependent differences, in-cell western blot analysis was performed to assess ERK/MAPK pathway activation. As shown in Fig. 2D and **E**, phosphorylated ERK (p-ERK) levels were significantly higher in hNPCs cultured on Matrigel and collagen IV compared with those on PLO, laminin, and PLO/laminin, while total ERK1/2 levels remained relatively consistent across groups. These findings suggest that collagen IV, similar to Matrigel, promotes hNPC proliferation and adhesion through enhanced ERK/MAPK signaling activation.

### Substrate-dependent regulation of hNPC viability and progenitor marker expression

**Fig. 3:**
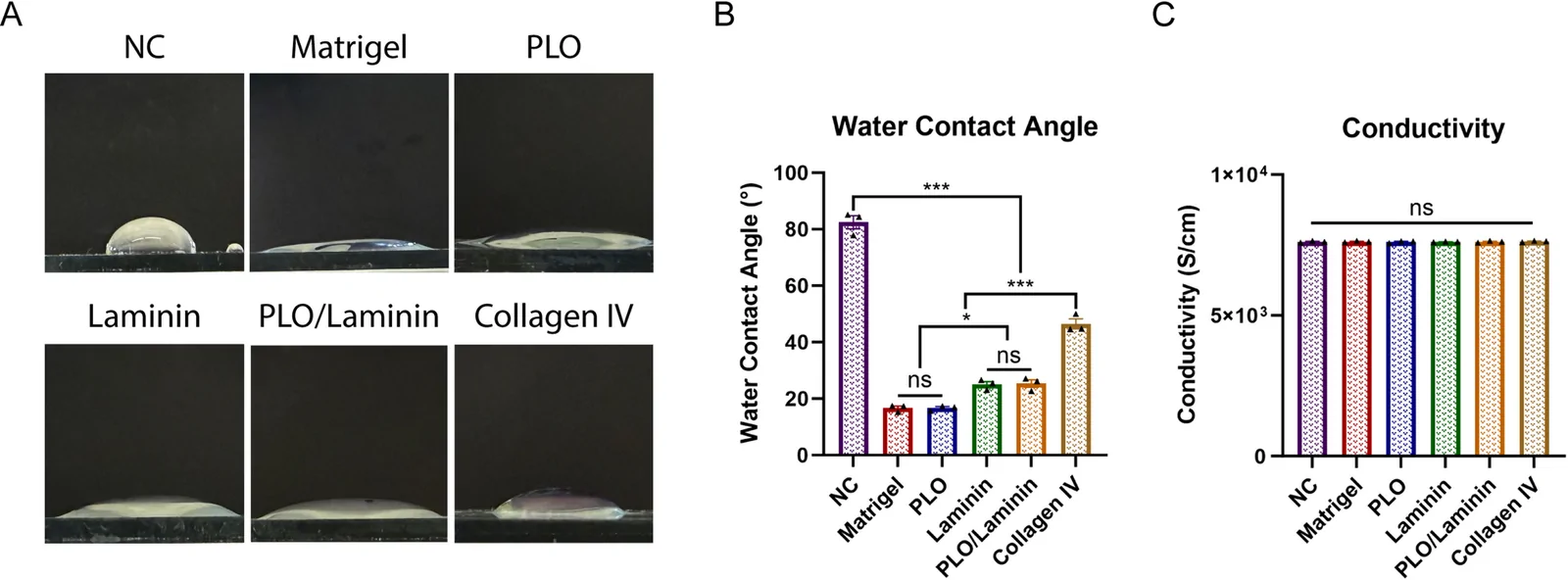
Surface wettability and electrical conductivity of ITO substrates coated with different extracellular matrix materials. (**A**) Representative optical images of water contact angle measurements on uncoated ITO glass (NC) and ITO glass coated with Matrigel, poly-L-ornithine (PLO), laminin, PLO/laminin, and collagen IV. (**B**) Quantitative analysis of water contact angles. (**C**) Electrical conductivity of ITO glass before and after surface coating. Data are presented as mean ± SEM. Statistical significance is indicated as *p < 0.05, **p < 0.01, ***p < 0.001; ns, not significant.

To assess whether hNPCs could survive and maintain viability on conductive neural interfaces, indium tin oxide (ITO) glass was covered with the various coatings and characterized prior to cell culture. Surface hydrophilicity was evaluated using water contact angle measurements. As shown in Fig. 3A and B, all coatings substantially improved the hydrophilicity of bare ITO glass. Among them, Matrigel- and PLO-coated surfaces exhibited the lowest contact angles, whereas collagen IV showed a moderately higher contact angle, indicating slightly reduced hydrophilicity relative to the other coatings. Importantly, electrical conductivity measurements demonstrated that none of the substrate coatings significantly altered the conductive properties of the ITO glass (Fig. 3C), confirming that the coatings preserved the electrical functionality of the conductive surface.

**Fig. 4:**
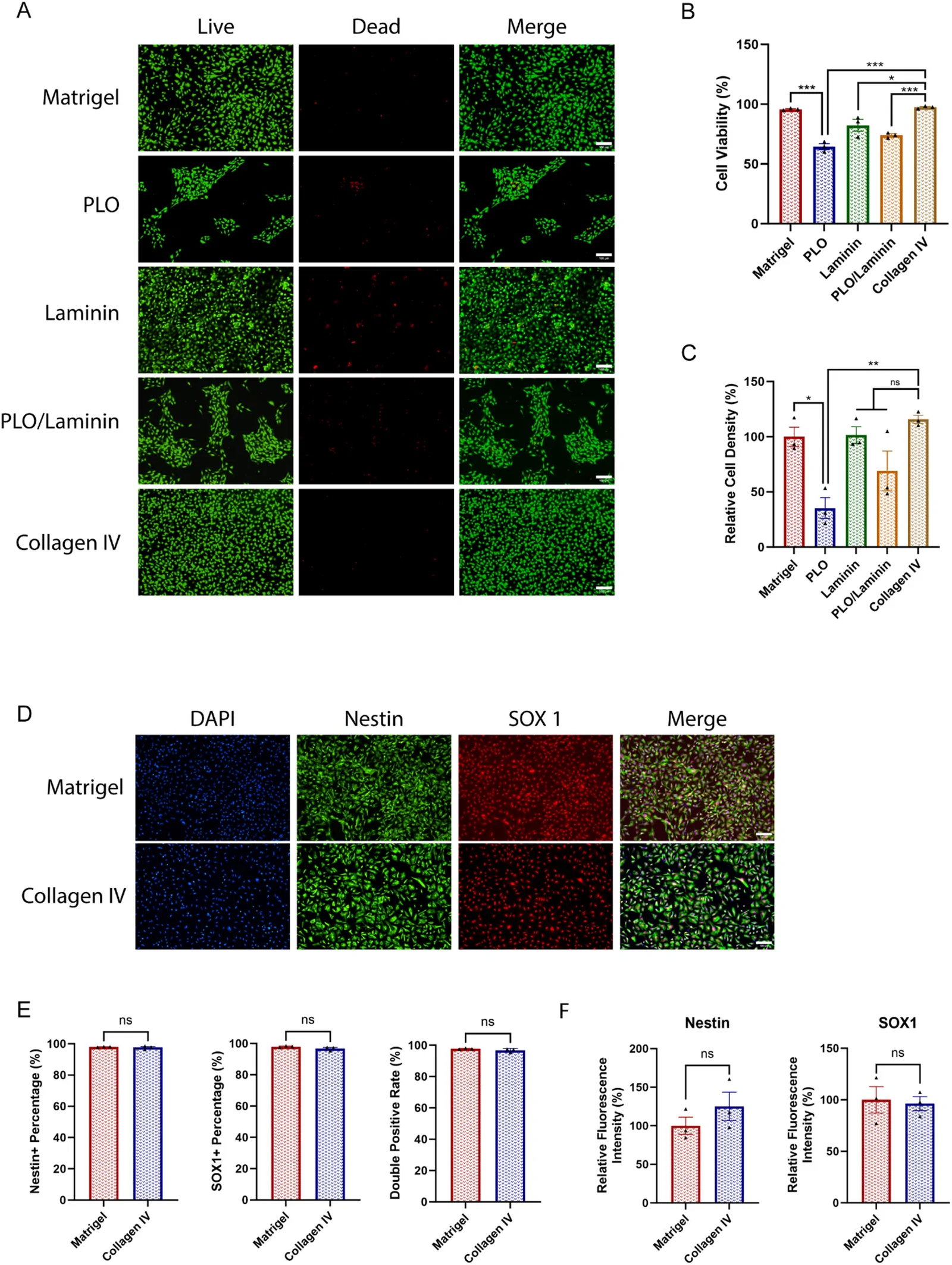
Substrate-dependent effects on hNPC viability and maintenance of progenitor characteristics. (**A**) Representative live/dead staining images of hNPCs on Matrigel, poly-L-ornithine (PLO), laminin, PLO/laminin, and collagen IV. Live cells are shown in green and dead cells in red. (**B**) Quantification of cell viability expressed as the percentage of live cells for each substrate. (**C**) Relative cell density of hNPCs cultured on different substrates. (**D**) Representative immunofluorescence images of hNPCs cultured on Matrigel and collagen IV, stained for nuclei (DAPI, blue), Nestin (green), and SOX1 (red). (**E**) Quantification of Nestin⁺, SOX1⁺, and Nestin⁺/SOX1⁺ double-positive hNPCs on Matrigel- and collagen IV–coated ITO substrates. (**F**) Relative fluorescence intensity of Nestin and SOX1 in hNPCs cultured on Matrigel and collagen IV. Data are presented as mean ± SEM. Statistical significance is indicated as *p < 0.05, **p < 0.01, ***p < 0.001; ns, not significant. Scale bars = 100 μm.

Live/dead staining was performed to further evaluate the cell behavior on conductive surface (Fig. 4A). Across all conditions, most cells remained viable, with sparse dead cells observed. Quantitative analysis revealed that hNPCs cultured on Matrigel and collagen IV exhibited the highest cell viability, both approaching 98% (Fig. 4B). In contrast, cells cultured on PLO and showed significantly reduced viability compared with Matrigel, while laminin and PLO/laminin supported intermediate levels of cell survival. Notably, collagen IV demonstrated significantly higher viability than PLO and PLO/laminin. Relative cell density analysis further revealed substrate-dependent differences in hNPC growth (Fig. 4C). Cells cultured on collagen IV exhibited the highest relative cell density, significantly exceeding that of PLO and PLO/laminin, while no significant difference was observed between collagen IV and Matrigel. These results indicate that collagen IV effectively supports both hNPC survival and cell expansion.

To assess whether collagen IV maintains hNPC progenitor characteristics comparable to Matrigel, immunofluorescence staining for Nestin and SOX1 was performed (Fig. 4D). Cells cultured on both substrates showed robust expression of Nestin and SOX1 with similar spatial distribution and morphology. Quantitative analysis demonstrated no significant difference in the percentage of Nestin⁺ or SOX1⁺ cells between Matrigel and collagen IV groups, and the percentage of Nestin⁺/SOX1⁺ double-positive cells was also comparable between the two groups, further supporting maintenance of the hNPC progenitor phenotype on collagen IV-coated conductive substrates (Fig. 4E). In addition, fluorescence intensity analysis showed comparable expression levels of Nestin and SOX1 on the two substrates, with no statistically significant differences observed (Fig. 4F). Collectively, these results demonstrate that collagen IV supports high hNPC viability, promotes increased cell density, and preserves progenitor marker expression at levels comparable to Matrigel.

### Maintenance of hNPC Viability and Progenitor Identity on Conductive ITO Substrates under Electrical Stimulation

**Fig. 5:**
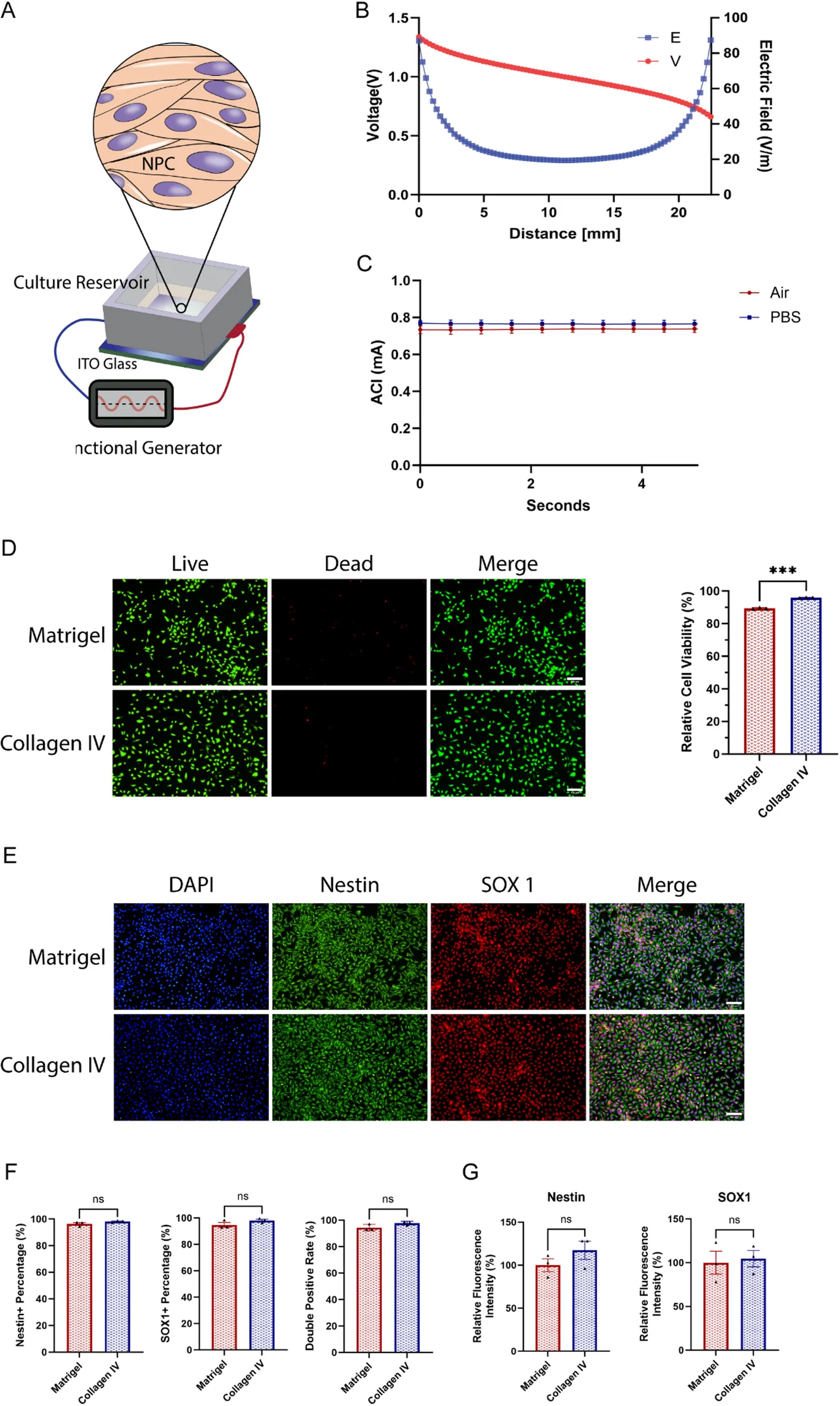
hNPC viability and maintenance of progenitor phenotype on conductive substrates under electrical stimulation. (**A**) Schematic illustration of the electrical stimulation culture system, showing hNPCs cultured on coated indium tin oxide (ITO) glass connected to a functional generator through a culture reservoir. (**B**) Finite element simulation of the electrical stimulation setup, illustrating electric field (E) and voltage (V) distributions across the conductive surface, indicating a stable and spatially uniform stimulation profile. (**C**) Current measurements along the ITO stimulation pathway under dry air and PBS-immersed conditions, showing comparable current delivery under culture-relevant aqueous conditions. (**D**) Representative live/dead staining images of hNPCs cultured on electrically stimulated ITO glass coated with Matrigel or collagen IV. Live cells are shown in green and dead cells in red. Quantification of relative cell viability of hNPCs cultured on Matrigel- and collagen IV–coated conductive substrates under electrical stimulation. (**E**) Representative immunofluorescence images of hNPCs cultured on conductive substrates, stained for nuclei (DAPI, blue), Nestin (green), and SOX1 (red). (**F**) Quantification of Nestin⁺, SOX1⁺, and Nestin⁺/SOX1⁺ double-positive hNPCs on conductive substrates. (**G**) Relative fluorescence intensity of Nestin and SOX1 in hNPCs cultured on conductive substrates. Data are presented as mean ± SEM. Statistical significance is indicated as *p < 0.05, **p < 0.01, ***p < 0.001; ns, not significant. Scale bars = 100 μm.

To evaluate the feasibility of culturing hNPCs on conductive substrates under electrical stimulation, a customized stimulation platform was established using ITO glass as the conductive surface (Fig. 5A). Finite element simulation demonstrated a spatially uniform electric field distribution across the culture area at around 20 V/m, with a gradual voltage drop along the stimulation direction (Fig. 5B), indicating stable and controllable electrical stimulation conditions. To further confirm electrical delivery through the ITO-based stimulation platform, current measurements were performed along the stimulation pathway under the same stimulation parameters used for cell culture experiments. A square-wave signal of 2 V peak-to-peak (Vpp) at 100 Hz was applied across the ITO surface. Measurements were conducted on dry ITO glass and on ITO glass immersed in PBS to mimic the aqueous culture environment. Comparable current values were detected under both conditions, indicating that immersion in PBS did not substantially attenuate current transmission through the ITO stimulation pathway (Fig. 5C). These results experimentally confirm that the conductive ITO substrate remained electrically active under culture-relevant conditions and support effective electrical delivery during hNPC stimulation experiments.

Live/dead staining revealed that hNPCs cultured on electrically stimulated ITO surfaces coated with either Matrigel or collagen IV maintained high viability, with minimal dead cells observed in both groups. Quantitative analysis confirmed that cell viability on collagen IV–coated ITO was slightly but significantly higher than that on Matrigel (Fig. 5D). Immunofluorescence staining further demonstrated robust expression of the neural progenitor markers Nestin and SOX1 in hNPCs cultured on both Matrigel- and collagen IV–coated conductive substrates (Fig. 5E). Quantitative analysis showed no significant differences in the percentage of Nestin⁺ or SOX1⁺ cells between the two groups. Importantly, analysis of Nestin⁺/SOX1⁺ double-positive cells also showed no significant difference between Matrigel and collagen IV, indicating that collagen IV preserved the core progenitor marker profile at a level comparable to Matrigel under electrical stimulation (Fig. 5F), nor in the relative fluorescence intensity of either marker (Fig. 5G). These results indicate that collagen IV supports hNPC survival and preserves progenitor identity on conductive surfaces under electrical stimulation, performing comparably to Matrigel while maintaining high cell viability.

## Discussion

hNPCs have been widely investigated in neural repair due to their dual functionality, they contribute directly to regeneration through multi-lineage differentiation into neurons, astrocytes, and oligodendrocytes, and indirectly through paracrine signaling, including the secretion of neurotrophic factors such as brain-derived neurotrophic factor (BDNF) and neurotrophin-3 (NT-3), which promote axonal growth, neuronal survival, and functional recovery^27^. Moreover, transplanted neural progenitor cells have been shown to enhance tissue repair by modulating the local microenvironment, supporting host cell survival, and facilitating synaptic integration^28^.

Matrigel remains a widely adopted standard for neural progenitor culture because it provides a basement-membrane-like milieu rich in ECM proteins and bioactive factors. However, its tumor-derived, xenogeneic origin introduces fundamental limitations for clinical translation, including ill-defined composition, batch-to-batch variability, and concerns regarding xenogeneic contaminants and regulatory acceptance for human implantation. These constraints are increasingly recognized across stem cell and organoid fields as major barriers to reproducibility and therapeutic manufacturing, fueling the push toward defined, animal-free, and tunable matrices^7,9,29^. Across our substrate screening experiments, collagen IV emerged as the most consistent Matrigel-alternative among the tested candidates (PLO, laminin, PLO/laminin, collagen IV). Although certain collagen-rich environments have been associated with inhibitory effects in neural regeneration, the inhibitory roles described in the spinal cord and peripheral nerve injury literature are primarily attributed to fibrillar collagens such as collagen I and III, that accumulate within the fibrotic scar and form a dense extracellular matrix barrier that impedes axonal regrowth^30,31^. In contrast, collagen IV is a network-forming basement membrane protein with a fundamentally different biological role. In this study, we revealed that Collagen IV supported high hNPC viability and, importantly, promoted higher relative cell density compared with the other defined coatings, aligning more closely with Matrigel. These outcomes are biologically plausible because collagen IV is a principal structural constituent of basement membranes and is also a major component within Matrigel itself^7,32^. Functionally, collagen IV can provide integrin-binding cues that couple adhesion to survival and proliferative signaling, thereby supporting sustained culture without requiring the complex, variable growth factor milieu present in Matrigel^12,33^. Moreover, recent work continues to show that specific ECM proteins, including collagen IV, can substantially influence neural-lineage cell morphology, growth, and trophic behavior, reinforcing the value of reductionist, single-component ECM studies for identifying defined culture conditions^6,34^.

Immunofluorescence analysis demonstrated that collagen IV effectively preserved the expression of canonical hNPC progenitor markers, Nestin and SOX1, at levels comparable to those observed on Matrigel, whereas cells cultured on PLO/laminin exhibited reduced marker expression and pronounced morphological alterations. Notably, the PLO and PLO/laminin group also showed weaker cell adhesion, lower cell density, and less stable spreading, suggesting that the observed decrease in progenitor marker expression may arise from suboptimal cell–substrate interactions rather than a controlled differentiation process. PLO-based coatings primarily support cell attachment through nonspecific electrostatic interactions, and the bioactivity of laminin in such systems is highly dependent on its presentation and surface adsorption. Inadequate integrin engagement under these conditions may limit the activation of adhesion-dependent signaling pathways required for maintaining progenitor identity, thereby contributing to altered cell state^33^. These observations underscore that cell viability alone is an insufficient metric for evaluating substrate performance, as compromised progenitor identity or aberrant morphology may predispose cells toward altered or undesirable differentiation trajectories. This consideration is particularly important in the context of standardized cell manufacturing for regenerative medicine, where defined and reproducible culture substrates are essential to ensure consistency and translational reliability^35,36^. Consistent with this paradigm, parallel advances in human pluripotent stem cell culture have established recombinant extracellular matrix proteins, such as laminin-511/521 and vitronectin, as effective xeno-free alternatives to Matrigel in chemically defined systems^37–39^. Collectively, these developments highlight that the replacement of Matrigel with defined substrates is not a niche methodological refinement but rather a central objective in clinically oriented stem cell engineering, including neural progenitor cell culture.

Mechanistically, our in-cell Western results showed that p-ERK/ERK levels were higher in hNPCs cultured on Matrigel and collagen IV compared with PLO, laminin, and PLO/laminin, paralleling the substrates that better supported cell attachment, spreading, and cell density. This relationship is consistent with long-standing evidence that cell spreading can activate MAPK signaling, linking ECM recognition to downstream programs that regulate survival and growth^40,41^. Activation of the ERK/MAPK pathway has been shown to regulate key processes involved in cell proliferation and survival, including transcription of growth-related genes and suppression of anoikis following ECM attachment^42,43^. Accordingly, ERK/MAPK signaling serves as a functional, pathway-level indicator of productive cell–substrate interactions, distinguishing substrates that elicit biologically active adhesion from those that merely enable nonspecific attachment^41,44^. Consistent with this framework, our results show that collagen IV preserve high level of ERK/MAPK activation alongside cell adhesion and spreading, whereas PLO and PLO/laminin substrates exhibit reduced signaling and less stable cell attachment. These observations reinforce that collagen IV provides a biologically instructive interface, supporting hNPC survival and expansion under the tested conditions.

We further evaluated substrate performance on conductive ITO glass, both under static conditions and electrical stimulation, which is clinically and technologically relevant because electrical stimulation is a commonly used modality in spinal cord injury (SCI) and peripheral nerve injury (PNI) care, applied to support functional recovery and rehabilitation^45,46^. For PNI specifically, contemporary clinical and translational literature supports electrical stimulation as a strategy that can enhance axonal regeneration and accelerate sensorimotor recovery, while ongoing research continues to refine stimulation parameters and mechanistic understanding^47,48^. In SCI, epidural spinal cord stimulation has shown meaningful functional improvements across multiple clinical reports and reviews, further underscoring the importance of integrating electrical cues into next-generation neural repair platforms^49,50^.

Our surface characterization data indicates that ECM coatings increased the hydrophilicity of ITO substrates without compromising electrical conductivity, an important enabling result given that surface modifications intended to improve cell adhesion can inadvertently disrupt electrical properties and undermine stimulation-based applications. The preservation of conductivity suggests that the coatings employed here are compatible with electrically active platforms and suitable for workflows requiring predictable electrical field delivery.

The electrical stimulation condition used in this study was selected to fall within commonly reported ranges for in vitro neural stimulation. Electric field strengths on the order of 10–100 V/m and frequencies from 1 to 100 Hz have been reported to influence neural progenitor cell behavior and maturation^21^. In addition, current measurements along the ITO stimulation pathway confirmed that electrical stimulation was successfully delivered under PBS-immersed, culture-relevant conditions. hNPCs cultured on collagen IV coated ITO maintained high viability and robust expression of progenitor markers, including under electrical stimulation, with viability modestly exceeding that observed on Matrigel coated substrates. Although collagen IV exhibited slightly lower surface hydrophilicity than PLO or laminin coatings, enhanced hNPC attachment and survival on collagen IV can be attributed to the quality of cell–substrate interactions rather than surface wettability alone. While hydrophilicity primarily influences initial, nonspecific cell attachment, sustained adhesion and proliferation of neural progenitor cells depend predominantly on integrin-mediated ligand recognition and downstream signaling^51,52^. As a native basement membrane component, collagen IV engages integrins such as α1β1 and α2β1, promoting focal adhesion maturation, cytoskeletal organization, and activation of survival-associated pathways, including ERK/MAPK signaling^53^. In contrast, highly hydrophilic polycationic coatings such as PLO rely largely on electrostatic interactions and lack defined biochemical cues required for stable integrin signaling, rendering cells more susceptible to stress or detachment^54,55^. Moreover, despite its comparatively lower hydrophilicity, collagen IV provides a biologically instructive and mechanically stable interface that supports hNPC viability and progenitor maintenance on conductive substrates, particularly in the presence of electrical stimulation where robust cell–ECM coupling is critical for maintaining cellular homeostasis^56^.

In summary, the present study seeks to replace Matrigel, an EHS sarcoma derived murine matrix unsuitable for direct human implantation, with a more defined ECM substrate capable of maintaining hNPC viability and progenitor identity, including in conductive and electrically stimulated environments. By demonstrating that collagen IV supports hNPC culture comparably to Matrigel and remains compatible with electrical stimulation paradigms used in SCI and PNI therapy, our findings provide a practical foundation for developing translatable, electrically active neural repair platforms.

## Materials and methods

### Preparation of different coating substrates

Coating solutions were prepared by diluting Matrigel (1:200, Corning, 356230, Lot #19323002) in Dulbecco’s Modified Eagle’s Medium/Ham’s F-12 (DMEM/F12, 50:50), poly-L-ornithine (PLO, 0.01 mg/ml, R&D Systems, 3436-100-01), laminin (0.01 mg/ml, Millipore, CC095), and collagen IV (5 µg/cm², Millipore, C5533) in phosphate-buffered saline (PBS). Tissue culture plates (TCPs, Genesee Scientific, 25–105) or glass coverslips were incubated with coating solutions at volumes of 1 mL per well for 6-well plates and 300 µL per well for 24-well plates. TCPs coated with Matrigel were incubated at 37 °C for 30 min, whereas substrates coated with PLO, laminin, PLO/laminin, or collagen IV were incubated at 37 °C overnight. Following incubation, all substrates were rinsed once with PBS prior to cell seeding.

### Neural progenitor cell differentiation and maintenance

Healthy human induced pluripotent stem cells (hiPSCs; JCAZ001, male) were derived from blood and purchased from the iPSC core of University of Arizona for non-commercial purposes, and all protocols were approved by the Institutional Biosafety Committee of University of Arizona. hNPCs were further differentiated as previously described^57^. Briefly, iPSCs were maintained in mTeSR basal medium (Stemcell Technologies) for 3 days. Upon reaching approximately 80% confluence, differentiation was initiated using a neural induction medium composed of 48% DMEM/F12, 48% Neurobasal medium (Gibco), 2% B27 supplement (Gibco), 1% N-2 MAX supplement (R&D Systems), 1% non-essential amino acids (NEAA, Cytiva), 1% GlutaMAX (Thermo Fisher Scientific), 1 µM dorsomorphin (Millipore), and 0.1 µM SB431542 (Millipore). Cells were cultured in this differentiation medium for 10 days. Following differentiation, cells were subcultured onto newly coated TCPs and maintained in hNPC maintenance medium consisting of 48% DMEM/F12, 48% Neurobasal medium, 2% B27, 1% N-2 MAX, 1% NEAA, 1% GlutaMAX, 20 ng/mL basic fibroblast growth factor (bFGF, R&D Systems), and 20 ng/mL epidermal growth factor (EGF, R&D Systems). hNPCs between passages 1 and 3 were used for all experiments. All cell cultures were maintained at 37 °C in a humidified incubator with 5% CO₂.

### Wettability test

Surface wettability of coated and uncoated ITO glass was evaluated using static water contact angle measurements. Briefly, ITO glass substrates were cleaned, coated with Matrigel, PLO, laminin, PLO/laminin, or collagen IV following the same procedures used for cell culture experiments, and allowed to equilibrate at room temperature prior to testing. Uncoated ITO glass was used as negative control. A droplet of deionized (DI) water was gently dispensed onto the substrate surface using a precision micropipette, ensuring minimal impact force to avoid droplet deformation. Optical images of the water droplet were captured immediately after deposition using a contact angle goniometer or a calibrated optical imaging system. The static contact angle was determined by fitting the droplet profile using Imagej. For each substrate condition, measurements were performed at a minimum of three randomly selected locations per sample, and at least three independent samples were analyzed. Lower contact angle values were interpreted as increased surface hydrophilicity.

### Conductivity test

The electrical conductivity of indium tin oxide (ITO) glass before and after surface coating was measured using a four-point probe tester (ST-2258 C) to minimize contact resistance effects. ITO glass substrates were coated with Matrigel, poly-L-ornithine (PLO), laminin, PLO/laminin, or collagen IV following the same procedures used for cell culture experiments. Uncoated ITO glass served as the control group. All samples were air-dried at room temperature prior to measurement. Conductivity measurements were performed by placing the four probes in linear contact with the sample surface under a constant applied current, and the resulting voltage drop between the inner probes was recorded. Sheet resistance values were calculated according to the standard four-point probe method. Electrical conductivity was subsequently derived from the measured sheet resistance based on the known thickness of the ITO layer provided by the manufacturer. For each substrate condition, measurements were taken at a minimum of three distinct locations on each sample, with at least three independent samples analyzed per group.

### Current measurement on ITO surface

To experimentally validate electrical delivery through the ITO stimulation platform, current measurements were performed under the same stimulation parameters used for cell culture experiments. ITO glass substrates were connected to the waveform generator (KEYSIGHT, EDU33212A). A square-wave electrical signal of 2 Vpp at 100 Hz was applied across the ITO surface, and the resulting current was measured using a digital multimeter (KEYSIGHT, 34465 A) connected in series with the stimulation circuit. Measurements were performed under dry ITO glass and ITO glass immersed in PBS, to mimic the conductive aqueous environment present during cell culture. For the PBS condition, sufficient PBS was added to cover the ITO surface while maintaining the same electrode spacing and contact configuration. Current values were recorded during the stimulation process.

### Electrical stimulation of NPCs

Electrical stimulation (ES) was applied to hNPCs cultured on ITO glass substrates using a custom two-electrode configuration. Briefly, coated ITO substrates were placed in sterile culture plates and two conductive probes were positioned on the ITO surface to serve as electrical contacts. The probes were connected to a function generator, which delivered a square waveform with an output amplitude of 2 Vpp at a frequency of 100 Hz. The applied stimulation parameters were designed to generate a uniform electric field across the ITO surface. Finite element simulations were performed using ANSYS software to model the electric field distribution based on the electrode configuration and applied voltage. Simulation results confirmed that the stimulation conditions produced a spatially uniform electric field with an average field strength of approximately 20 V/m at the cell culture surface (Fig. 5A, B). ES was applied for 10 min per day throughout the designated stimulation period of 5 days. During stimulation, cells were maintained in standard culture medium under routine incubator conditions except for the brief stimulation interval.

### Alamar blue assay

hNPCs were seeded in 96-well plates at a density of 5,000/well, and the experiment was performed using AlamarBlue cell viability reagent (ThermoFisher Scientific) on day 1, 3 and 5, respectively, following the manufacturer’s instructions. Briefly, a working solution was prepared by diluting Alamar blue reagent (1:10) with hNPC maintenance medium. Discard the medium in 96-well plate and rinse the cells with PBS once, then working solution was added to each well, and wells without cells were applied as background. The TCPs were then incubated at 37℃ for 4 h. A plate reader was used to record the absorbance of each sample at 570 nm and the background at 600 nm, and the cell viability was calculated through: $\:Cell\:Viability=\frac{{Ab}_{570}-{Ab}_{600}}{{Ab}_{Control}-{Ab}_{600}}$, and the Matrigel group was determined as control group.

### Cell live/dead staining

NPCs were seeded on cover slips or ITO glass, and live/dead staining was performed according to the manufacturer’s instruction. Briefly, the cells were treated with staining solution containing calcein AM (ThermoFisher Scientific) and ethidium homodimer-1 (Millipore Sigma), and cells were then incubated for 20 min at room temperature. After rinsing with PBS, images were taken using fluorescent microscope. At least 3 fields were taken for each sample.

### Immunofluorescent staining

NPCs were seeded on cover slips or ITO glass, the culture medium was removed, and the cells were fixed using 4% paraformaldehyde for 15 min at room temperature. Following fixation, the cells were permeabilized with 0.1% Triton X-100 for 10 min at room temperature after the formaldehyde was removed. 1% bovine serum albumin (BSA) was then applied for blocking, and the cells were incubated for 1 h at room temperature. The cells were subsequently treated with primary antibodies and incubated overnight at 4 °C. The following day, the antibodies were removed, and the cells were washed with PBS for 5 min, and this process was repeated three times. Afterward, secondary antibodies were introduced, and the cells were incubated for 1 h at room temperature. Cells were then rinsed three times with PBS and then incubated in 300 nM DAPI solution for 10 min at room temperature. Images were then taken following three times wash with PBS using fluorescent microscope (Nikon Eclipse Ti2). Antibodies applied in this study were as follows: Nestin (Millipore, ABD69), SOX1 (R&D Systems, AF3369), Donkey anti-Rabbit IgG (H + L) Highly Cross-Adsorbed Secondary Antibody (Fisher Scientific, PIA32790), Donkey anti-Goat IgG (H + L) Highly Cross-Adsorbed Secondary Antibody (Fisher Scientific, PIA32758), DAPI and Hoechst Nucleic Acid Stains (Fisher Scientific, D1306).

### In-cell western blot

NPCs were seeded in 96-well plates at a density of 10,000/cm^2^, the culture medium was removed, and the cells were fixed using 3.7% formaldehyde for 20 min at room temperature. Following fixation, the cells were permeabilized with 0.2% Triton X-100 for 10 min at room temperature after the formaldehyde was removed. A blocking buffer (Intercept PBS) was then applied, and the cells were incubated for 1.5 h at room temperature. The cells were subsequently treated with primary antibodies and incubated overnight at 4 °C. The following day, the antibodies were removed, and the cells were washed with 0.1% phosphate-buffered saline containing Tween 20 (PBST) for 5 min, and this process was repeated five times. Afterward, CellTag 520 and secondary antibodies were introduced, and the cells were incubated for 1 h at room temperature. Cells were rinsed five times with 0.1% PBST, and imaging was conducted using the Odyssey M Imager at wavelengths of 520, 700, and 800 nm, respectively. The results were normalized to the absorbance measured at 520 nm. p-ERK levels were normalized to total ERK and cell-associated signal. Antibodies applied in this study were as follows: ERK 1/2 Antibody (C-9) (Santa Cruz Biotechnology, sc-514302); Phospho-p44/42 MAPK (Erk1/2) (Thr202/Tyr204) Antibody (Cell Signaling Technology, 9101 S); IRDye^®^ 680RD Goat anti-Mouse IgG Secondary Antibody (Li-COR Biosciences, 926-68070); IRDye^®^ 800CW Goat anti-Rabbit IgG Secondary Antibody, 1:200(Li-COR Biosciences, 926-32211); CellTag 520 stain (Li-COR Biosciences, 926-41094).

### Statistical analysis

All quantitative data are presented as mean ± standard error of the mean (SEM). Statistical analyses were performed using GraphPad Prism (GraphPad Software, USA). For comparisons involving more than two groups, one-way analysis of variance (ANOVA) was applied, followed by Tukey’s post hoc multiple-comparison test to identify statistically significant differences between individual groups. For direct comparisons between two groups, an unpaired two-tailed Student’s T-test was used. Each experiment was independently repeated at least three times unless otherwise stated. Statistical significance was defined as *p* < 0.05.

## Data Availability

The data presented in this study are openly available in this article. Additional data is available from the corresponding author upon request.

## References

[1] Yousefifard, M. et al. Neural stem/progenitor cell transplantation for spinal cord injury treatment; A systematic review and meta-analysis. *Neuroscience* **322**, 377–397 (2016). 26917272 10.1016/j.neuroscience.2016.02.034

[2] Rayner, M. L. D., Day, A. G. E., Bhangra, K. S., Sinden, J. & Phillips, J. B. Engineered neural tissue made using clinical-grade human neural stem cells supports regeneration in a long gap peripheral nerve injury model. *Acta Biomater.* **135**, 203–213 (2021). 34455110 10.1016/j.actbio.2021.08.030

[3] Yi, S. et al. Application of stem cells in peripheral nerve regeneration. *Burns Trauma.* **8**, tkaa002 (2020). 32346538 10.1093/burnst/tkaa002 PMC7175760

[4] Yang, K. et al. Polydopamine-mediated surface modification of scaffold materials for human neural stem cell engineering. *Biomaterials* **33**, 6952–6964 (2012). 22809643 10.1016/j.biomaterials.2012.06.067

[5] Discher, D. E., Janmey, P. & Wang, Y. Tissue Cells Feel and Respond to the Stiffness of Their Substrate. *Science* **310**, 1139–1143 (2005). 16293750 10.1126/science.1116995

[6] Saha, K. et al. Substrate Modulus Directs Neural Stem Cell Behavior. *Biophys. J.* **95**, 4426–4438 (2008). 18658232 10.1529/biophysj.108.132217 PMC2567955

[7] Benton, G. et al. From discovery and ECM mimicry to assays and models for cancer research. *Adv. Drug Deliv. Rev.* **79–80**, 3–18 (2014). 24997339 10.1016/j.addr.2014.06.005

[8] Passaniti, A., Kleinman, H. K. & Martin, G. R. Matrigel: history/background, uses, and future applications. *J. Cell. Commun. Signal.* **16**, 621–626 (2022). 34463918 10.1007/s12079-021-00643-1 PMC9733768

[9] Aisenbrey, E. A. & Murphy, W. L. Synthetic alternatives to Matrigel. *Nat. Rev. Mater.* **5**, 539–551 (2020). 32953138 10.1038/s41578-020-0199-8 PMC7500703

[10] Luckenbill-Edds, L. Laminin and the mechanism of neuronal outgrowth. *Brain Res. Rev.* **23**, 1–27 (1997). 9063584 10.1016/s0165-0173(96)00013-6

[11] Jansson, L. C. & Åkerman, K. E. The role of glutamate and its receptors in the proliferation, migration, differentiation and survival of neural progenitor cells. *J. Neural Transm*. **121**, 819–836 (2014). 24562403 10.1007/s00702-014-1174-6

[12] Li, H. Y. et al. Collagen IV Significantly Enhances Migration and Transplantation of Embryonic Stem Cells: Involvement of α2β1 Integrin-Mediated Actin Remodeling. *Cell. Transpl.* **20**, 893–908 (2011). 10.3727/096368910X550206 21176409

[13] Ali, S. A., Pappas, I. S. & Parnavelas, J. G. Collagen type IV promotes the differentiation of neuronal progenitors and inhibits astroglial differentiation in cortical cell cultures. *Dev. Brain Res.* **110**, 31–38 (1998). 9733911 10.1016/s0165-3806(98)00091-1

[14] Li, S., Liu, Y., Luo, X. & Hong, W. Systematic Evaluation of Extracellular Coating Matrix on the Differentiation of Human-Induced Pluripotent Stem Cells to Cortical Neurons. *Int. J. Mol. Sci.* **26**, 230 (2025). 10.3390/ijms26010230 PMC11720352 39796088

[15] Song, S. et al. Electrical stimulation of human neural stem cells via conductive polymer nerve guides enhances peripheral nerve recovery. *Biomaterials* **275**, 120982 (2021). 34214785 10.1016/j.biomaterials.2021.120982 PMC8325644

[16] Omer, S. A., McKnight, K. H., Young, L. I. & Song, S. Stimulation strategies for electrical and magnetic modulation of cells and tissues. *Cell. Regen*. **12**, 21 (2023). 37391680 10.1186/s13619-023-00165-8 PMC10313634

[17] Reynolds, M. et al. Fabrication of Sodium Trimetaphosphate-Based PEDOT:PSS Conductive Hydrogels. *Gels* 10, (2024). 10.3390/gels10020115 PMC10888113 38391444

[18] Li, L., Hernandez, A., Grevsmuehl, R., Kou, Y. T. & Song, S. Engineering 3D-printed standalone conductive nerve guides using soft bioinks for peripheral nerve injuries. *Mater. Adv.* **7**, 606–617 (2026). 41333609 10.1039/d5ma00932d PMC12666618

[19] Santhanam, S. et al. Controlling the Stem Cell Environment Via Conducting Polymer Hydrogels to Enhance Therapeutic Potential. *Adv. Mater. Technol.* **8**, 2201724 (2023). 42238535 10.1002/admt.202201724 PMC13229573

[20] Oh, B. et al. Electrical modulation of transplanted stem cells improves functional recovery in a rodent model of stroke. *Nat. Commun.* **13**, 1366 (2022). 35292643 10.1038/s41467-022-29017-w PMC8924243

[21] Cheng, H., Huang, Y., Yue, H. & Fan, Y. Electrical Stimulation Promotes Stem Cell Neural Differentiation in Tissue Engineering. *Stem Cells International* 6697574 (2021). (2021). 10.1155/2021/6697574 PMC8081629 33968150

[22] Zuo, K. J., Gordon, T., Chan, K. M. & Borschel, G. H. Electrical stimulation to enhance peripheral nerve regeneration: Update in molecular investigations and clinical translation. *Exp. Neurol.* **332**, 113397 (2020). 32628968 10.1016/j.expneurol.2020.113397

[23] Liu, Y. et al. Morphing electronics enable neuromodulation in growing tissue. *Nat. Biotechnol.* **38**, 1031–1036 (2020). 32313193 10.1038/s41587-020-0495-2 PMC7805559

[24] Li, L. et al. Direct-Current Electrical Field Guides Neuronal Stem/Progenitor Cell Migration. *Stem Cells*. **26**, 2193–2200 (2008). 18556511 10.1634/stemcells.2007-1022

[25] Chang, Y. J., Hsu, C. M., Lin, C. H., Lu, M. S. C. & Chen, L. Electrical stimulation promotes nerve growth factor-induced neurite outgrowth and signaling. *Biochim. et Biophys. Acta (BBA) - Gen. Subj.* **1830**, 4130–4136 (2013). 10.1016/j.bbagen.2013.04.007 23583367

[26] Song, S. et al. Conductive gradient hydrogels allow spatial control of adult stem cell fate. (2024). 10.1039/D3TB02269B doi:10.1039/D3TB02269B. 10.1039/d3tb02269b PMC10922832 38291979

[27] Wang, C., Lu, C., Peng, J., Hu, C. & Wang, Y. Roles of neural stem cells in the repair of peripheral nerve injury. *Neural Regeneration Res.* **12**, 2106 (2017). 10.4103/1673-5374.221171 PMC5784362 29323053

[28] Oh, B. et al. Modulating the Electrical and Mechanical Microenvironment to Guide Neuronal Stem Cell Differentiation. *Adv. Sci.* **8**, 2002112 (2021). 10.1002/advs.202002112 PMC8025039 33854874

[29] Wolff, L. & Hendrix, S. Rethinking Matrigel: The Complex Journey to Matrix Alternatives in Organoid Culture. *Adv. Sci.* **12**, e08734 (2025). 10.1002/advs.202508734 PMC12713094 41204762

[30] Soderblom, C. et al. Perivascular Fibroblasts Form the Fibrotic Scar after Contusive Spinal Cord Injury. *J. Neurosci.* **33**, 13882–13887 (2013). 23966707 10.1523/JNEUROSCI.2524-13.2013 PMC3755723

[31] Orr, M. B. & Gensel, J. C. Spinal Cord Injury Scarring and Inflammation: Therapies Targeting Glial and Inflammatory Responses. *Neurotherapeutics* **15**, 541–553 (2018). 29717413 10.1007/s13311-018-0631-6 PMC6095779

[32] Kleinman, H. K. et al. Isolation and characterization of type IV procollagen, laminin, and heparan sulfate proteoglycan from the EHS sarcoma. *ACS Publications*. 10.1021/bi00267a025 (2002). https://pubs.acs.org/doi/pdf/10.1021/bi00267a025 6217835

[33] Hynes, R. O. & Integrins Bidirectional, Allosteric Signaling Machines. *Cell* **110**, 673–687 (2002). 12297042 10.1016/s0092-8674(02)00971-6

[34] Flanagan, L. A., Ju, Y. E., Marg, B., Osterfield, M. & Janmey, P. A. Neurite branching on deformable substrates. *NeuroReport* **13**, 2411 (2002). 12499839 10.1097/01.wnr.0000048003.96487.97 PMC2408859

[35] Chaudhary, N. & Villa-Diaz, L. G. Advancements in the in vitro culture of human pluripotent stem cells:progress, challenges, and future directions: comprehensive review. *Front. Toxicol.* **7**, 1667573 (2025). 41409779 10.3389/ftox.2025.1667573 PMC12705378

[36] Costa, M. H. G., Carrondo, M. J. T., Alves, P. M. & Serra, M. Bridging process diversity in cell therapies toward standardization of manufacturing workflows. *Cytotherapy* **102041**10.1016/j.jcyt.2025.102041 (2026). 10.1016/j.jcyt.2025.102041 41762958

[37] Rodin, S. et al. Clonal culturing of human embryonic stem cells on laminin-521/E-cadherin matrix in defined and xeno-free environment. *Nat. Commun.* **5**, 3195 (2014). 24463987 10.1038/ncomms4195

[38] Rodin, S. et al. Long-term self-renewal of human pluripotent stem cells on human recombinant laminin-511. *Nat. Biotechnol.* **28**, 611–615 (2010). 20512123 10.1038/nbt.1620

[39] Braam, S. R. et al. Recombinant Vitronectin Is a Functionally Defined Substrate That Supports Human Embryonic Stem Cell Self-Renewal via αVβ5 Integrin. *Stem Cells*. **26**, 2257–2265 (2008). 18599809 10.1634/stemcells.2008-0291

[40] Giancotti, F. G. & Ruoslahti, E. *Integrin Signal. Science* **285**, 1028–1033 (1999). 10446041 10.1126/science.285.5430.1028

[41] Schwartz, M. A. & Ginsberg, M. H. Networks and crosstalk: integrin signalling spreads. *Nat. Cell. Biol.* **4**, E65–E68 (2002). 11944032 10.1038/ncb0402-e65

[42] Roskoski, R. ERK1/2 MAP kinases: Structure, function, and regulation. *Pharmacol. Res.* **66**, 105–143 (2012). 22569528 10.1016/j.phrs.2012.04.005

[43] Dhillon, A. S., Hagan, S., Rath, O. & Kolch, W. MAP kinase signalling pathways in cancer. *Oncogene* **26**, 3279–3290 (2007). 17496922 10.1038/sj.onc.1210421

[44] Chen, C. S., Mrksich, M., Huang, S., Whitesides, G. M. & Ingber, D. E. Geometric Control of Cell Life and Death. *Science* **276**, 1425–1428 (1997). 9162012 10.1126/science.276.5317.1425

[45] Hardy, P. B., Wang, B. Y., Chan, K. M., Webber, C. A. & Senger, J. L. B. The use of electrical stimulation to enhance recovery following peripheral nerve injury. *Muscle Nerve*. **70**, 1151–1162 (2024). 39347555 10.1002/mus.28262

[46] Luo, S., Xu, H., Zuo, Y., Liu, X. & All, A. H. A Review of Functional Electrical Stimulation Treatment in Spinal Cord Injury. *Neuromol Med.* **22**, 447–463 (2020). 10.1007/s12017-019-08589-9 31916220

[47] Willand, M. P., Nguyen, M. A., Borschel, G. H. & Gordon, T. Electrical Stimulation to Promote Peripheral Nerve Regeneration. *Neurorehabil Neural Repair.* **30**, 490–496 (2016). 26359343 10.1177/1545968315604399

[48] Juckett, L., Saffari, T. M., Ormseth, B., Senger, J. L. & Moore, A. M. The Effect of Electrical Stimulation on Nerve Regeneration Following Peripheral Nerve Injury. *Biomolecules* **12**, 1856 (2022). 36551285 10.3390/biom12121856 PMC9775635

[49] Taccola, G., Barber, S., Horner, P. J., Bazo, H. A. C. & Sayenko, D. Complications of epidural spinal stimulation: lessons from the past and alternatives for the future. *Spinal Cord*. **58**, 1049–1059 (2020). 32576946 10.1038/s41393-020-0505-8

[50] Hachmann, J. T. et al. Epidural spinal cord stimulation as an intervention for motor recovery after motor complete spinal cord injury. *J. Neurophysiol.* **126**, 1843–1859 (2021). 34669485 10.1152/jn.00020.2021

[51] Keselowsky, B. G., Collard, D. M. & García, A. J. Integrin binding specificity regulates biomaterial surface chemistry effects on cell differentiation. *Proceedings of the National Academy of Sciences* 102, 5953–5957 (2005). 10.1073/pnas.0407356102 PMC1087905 15827122

[52] Hersel, U., Dahmen, C. & Kessler, H. RGD modified polymers: biomaterials for stimulated cell adhesion and beyond. *Biomaterials* **24**, 4385–4415 (2003). 12922151 10.1016/s0142-9612(03)00343-0

[53] Sanders, M. A. & Basson, M. D. Collagen IV regulates Caco-2 migration and ERK activation via α1β1- and α2β1-integrin-dependent Src kinase activation. *Am. J. Physiology-Gastrointestinal Liver Physiol.* **286**, G547–G557 (2004). 10.1152/ajpgi.00262.2003 14604860

[54] Vroemen, P. A. M. M. et al. The Importance of Coating Surface and Composition for Attachment and Survival of Neuronal Cells Under Mechanical Stimulation. *J. Biomed. Mater. Res. A*. **113**, e37919 (2025). 40285752 10.1002/jbm.a.37919

[55] Hoshiba, T. et al. Regulation of the Contribution of Integrin to Cell Attachment on Poly(2-Methoxyethyl Acrylate) (PMEA) Analogous Polymers for Attachment-Based Cell Enrichment. *PLOS ONE*. **10**, e0136066 (2015). 26288362 10.1371/journal.pone.0136066 PMC4545787

[56] Humphrey, J. D., Dufresne, E. R. & Schwartz, M. A. Mechanotransduction and extracellular matrix homeostasis. *Nat. Rev. Mol. Cell. Biol.* **15**, 802–812 (2014). 25355505 10.1038/nrm3896 PMC4513363

[57] Song, S. et al. Controlling properties of human neural progenitor cells using 2D and 3D conductive polymer scaffolds. *Sci. Rep.* **9**, 19565 (2019). 31863072 10.1038/s41598-019-56021-w PMC6925212

